# Benefits and constraints of intimate partnerships for HIV positive sex workers in Kibera, Kenya

**DOI:** 10.1186/1475-9276-12-76

**Published:** 2013-09-03

**Authors:** Cecilia Benoit, Eric Roth, Helga Hallgrimsdottir, Mikael Jansson, Elizabeth Ngugi, Kimberly Sharpe

**Affiliations:** 1 Centre for Addictions Research of BC and Department of Sociology, University of Victoria, Victoria, Canada; 2 Department of Sociology, University of Victoria, Victoria, Canada; 3 Depart of Anthropology, University of Victoria, Victoria, Canada; 4 Centre for HIV Prevention and Research, University of Nairobi, Nairobi, Kenya

**Keywords:** Intimate partnerships, Female sex workers, HIV, Resource-constrained countries, Empowerment approach, Kenya

## Abstract

**Introduction:**

Research on the intimate partnerships of female sex workers (FSWs) tends to focus on the risks associated with these relationships. This paper takes as its starting point that the situation of FSWs is better understood by including knowledge of the benefits of their intimate partnerships. Specifically, we employ the conceptual framework provided by emergent research examining intimacy as a complex fusion of affective and instrumental dimensions among sex workers. This perspective allows us to frame information about FSWs’ intimate partnerships within a behaviour-structural approach that is helpful for identifying how intimate partnerships can be a source of both benefit as well as increased risk to FSWs.

**Methods:**

Our results are based on a mixed-methods study carried out in the summer of 2011 in Kibera, Kenya. We conducted face-to-face interviews (n=30) with a non-probability sample of FSWs stratified by age who self-identified as Human Immune Virus positive (HIV+). We asked about participants’ involvement in current and past intimate partnerships, and whether these relationships had a positive or negative impact on their health and well‒being.

**Results:**

Participants currently in intimate partnerships had fewer clients and thus lower incomes than those without intimate partnerships. Participants presently with partners were also more likely to receive some financial support from partners, to report lower intimate partner violence, and to narrate higher partner emotional support and greater assistance with medications. These participants were also more likely to have disclosed their sex work and HIV+ statuses to their partners. Intimate partnerships, on the other hand, showed increased risk of economic vulnerability and emotional dependence for FSWs. This became especially problematic for those participants in fragile relationships. Despite these variations, none of the differences between the two groups were statistically significant.

**Conclusions:**

Intimacy and transactional relations are bound up with one another and intersect with the structural realities and vulnerabilities; this is the case for sex workers in well-resourced and resourced-constrained countries alike. Rather than treating intimate partnerships as distinct from transactional relationships, FSWs’ relationships should be viewed on a continuum of risk and support.

## Introduction

Much of the scholarship on sex work is framed by an abolitionist approach that views the global sex industry as fundamentally coercive and exploitative of women [1]. Weitzer [2] rejects such binaries and instead advocates for research that sheds light on the intended and unintended effects of the structural environment on the health of sex workers.

One knowledge gap concerns potential benefits of intimacy between sex workers and their clients and with non-paying partners in private life. However, when “regular” or repeat clients and non-paying partners are mentioned in the literature, the usual focus is on the negative consequences of engagement, including that sex workers are less likely to use condoms with their regular clients or with intimate partners than with individuals who are unknown to them [3,4]. Lower condom use in intimate partnerships among FSWs likely has many causes; in contexts such as Kenya, lower condom use intersects with other cultural factors, such as negative associations between condom use, sexual pleasure and hegemonic notions of masculinity [5,6]. It is also linked to shared understandings between FSWs and their intimate partners that using a condom is indicative of a low level of trust in an intimate partnership, in a context where trust may emanate more from material support than sexual fidelity. Other studies have reported increased risk of physical violence in sex workers’ intimate partnerships in different regions of Africa [7,8]. As a result, intimacy is generally portrayed as harmful to sex workers’ health, lending support to the dominant moral view of prostitution as inherently exploitative and in need of punitive policies intended to eliminate the commercial sex industry [9,10].

A small number of studies have challenged this overriding perception of intimacy and sex work. In a study of FSWs working in the border provinces of Vietnam, Thuong et al. [11] found that having a non-paying intimate partner was a protective factor for the Human immunodeficiency virus (HIV). Likewise, Ngugi et al. [12] found that being in an intimate relationship decreased the number of clients FSWs served per week and increased condom use with paying clients; these findings were linked to the substantial financial contributions intimate partners made to the worker’s household income.

These studies demonstrate some potential benefits as well as harms of sex workers’ intimate partnerships. To understand both benefits and harms requires paying close attention to the responses of sex workers themselves as they speak about their work and relationships outside work. This study sheds light on the contributions of intimate partnerships to the health, safety, and well-being of a convenience sample of HIV+ FSWs residing in a large urban slum in Kenya. We frame our study within a behavioral–structural approach [13,14] positing that larger exploitative economic and social structures and lingering patriarchal policies influence unsafe commercial sex [15]. At the same time, the empowerment of FSWs, including their access to supportive intimate partnerships, reduces their exposure to health harms and promotes safety and well-being. As Blanchard et al. [16] note, empowerment strategies serve “as a mechanism whereby FSWs achieve the power to overcome this disadvantaged position and gain the agency to address their HIV vulnerability (p. 2)”. Before presenting the results of our study on the benefits and constraints of intimate partnerships in a specific resource-limited region, we summarize emergent conceptualizations of intimacy outside as well as inside sex work.

### Conceptualizing intimacy in human relationships

Definitions of intimacy suggest it is characterized by affection and some level of trust, as well as non-specificity in terms of the focus of interactions. Intimacy is often contrasted with instrumentality, which involves having a single focus (e.g., monetary payment) for one’s actions [17,18]. Intimate relationships are understood to be very strongly linked to individual physical health and welfare [19]. Research on intimate relationships suggests that partners tend to be happier than those not in such relationships, especially in recent decades marked by a decline in traditional patriarchal authority institutions [20]. Giddens [21] suggests that intimacy in ‘late modern’ well-resourced societies is more and more divorced from instrumentality; intimate relationships have become ‘unfettered’ from the overarching structural constraints (such as the economic dependence of wives upon husbands) that previously maintained and sustained relationships in traditional societies. Such ‘pure’ or ‘confluent’ relationships are centred around open communication and the satisfaction of emotional needs, and have great potential to lead to higher individual happiness, transformation of the gender order, and ultimately, the democratization of marriage. At the same time, according to Giddens [21], such relationships, which give individuals a great deal of choice, are unpredictable, and may engulf them with anxiety and direct them towards compensatory addictive behaviours, including chronic gambling and substance use.

Yet even in well-resourced countries intimate relationships remain circumscribed by cultural practices, dominant societal values and economic inequality [22]. While intimate partners increasingly use egalitarian discourses to *describe* their relationships, these relationships also remain ‘highly gendered’ and unequal in terms of care and housework, sex and money [23]. For instance, research on long distance relationships found that it is mostly women who tend “to sacrifice their individual desires and plans in order to look after loved ones” [24], p. 252.

Scholars have also drawn attention to the complex ways in which intimate relationships remain constituted simultaneously through affective and transactional relations. Zelizer argues that economic transactions abound within intimate relationships and are necessary for the functioning of households and ongoing negotiations between couples. People initiate and negotiate relationships and delineate boundaries “among the rights, obligations, transactions, and meanings that belong to ‘different’ social ties” [25]. Rather than existing on two separate planes, economic and affective dimensions continue to be fused within intimate relationships; as shown next, this fusion is illustrated through the prominence of negotiations around intimate or emotional services, in addition to sexual services, in interactions between sex workers and their clients in late modern societies.

### Intimacy and sex work in well-resourced countries

Recent scholarship in Euro-North America on intimacy in sex work complicates the division between economic and affective dimensions even more. Bernstein [26] uses the term ‘bounded authenticity’ to suggest that what is being sold and purchased between sex workers and clients in the United States today is an authentic emotional as well as physical relationship; yet this intimate exchange is temporarily and emotionally-bounded. An example of this is the ‘girlfriend experience’, where what is sold is a manufactured authenticity that tries to simulate being a girlfriend, involving communication of an unpretentious urge and sincere pleasure for a paying client who often seeks a ‘real’ and reciprocal, albeit delimited, sexual connection. The central point here is that emotional authenticity, especially in the context of late modernity, is understood to be explicitly part of sex work. Nevertheless, it is deeply implicated by economic reality: sex work encounters remain commercial affairs and there are no guarantees that sex worker and client will actually enjoy the company of one another. As Bernstein states: “Bounded authenticity lends itself less to engrossing time commitments or unwieldy rafts of dependents and more to intimate relations that are cost-efficient and well-suited to the structure of the modern corporation; temporary, detachable, and flexible” (p. 175).

This literature further underscores how categories of oppression/victimization versus empowerment cannot begin to capture the complex dynamics of commercial sex services [27-31]. In their study of street-level sex work in Vancouver, Canada, Shannon and colleagues [32] found a diversity of sex worker-intimate partner relationships. Some participants characterized their partners as “glorified pimps” because their intimate partners held significant power over their sex work encounters and controlled their access to resources, including drugs. Other participants stated that their intimate partners occupied a more traditional boyfriend role before transitioning into a pimp role to facilitate sex work for drugs and money. Yet even as ‘pimps’, these relationships were not considered solely exploitative: intimate partners were characterized as a continuing source of emotional support, companionship, trust, and as aids in resource acquisition. Similarly, Jackson et al. [33], in a study of street-based sex workers in Nova Scotia, Canada, found that intimate partners could be a source of social and emotional support. Some relationships represented a safe haven where women felt comfortable and accepted as themselves, and this fostered a feeling of inclusion. Other relationships reinforced outside stigma surrounding sex work, leading women to feel even further marginalized. This conclusion relates to the more general discussion about how sex workers negotiate personal spaces independent of their work, and it correlates to other studies mentioned above about the importance of marital and common-law intimate relationships support networks for both men and women’s health [34].

### Intimacy and sex work in less-resourced regions

Research conducted in countries outside Euro-North America on intimacy between sex workers and clients as well as with non-paying partners suggests more similarities than differences in practices of intimacy across cultures, calling into question assumptions linking particular forms of love and intimacy to late modern conditions peculiar to well-resourced countries. Studies from the Dominican Republic document how sex workers articulate their activities as efforts to get ahead (*progresar*), which sometimes culminate in love, marriage and possible immigration with their mostly European clients [35]. Other research on sex work in Caribbean resort towns shows there is a “general tendency to back away from overtly commodifying sexual relations”, with the purpose of preserving the dignity of the local participant [36]. In the case of Thailand, research suggests that most forms of sex work require previous social engagement and continuous involvement over time, although the level of economic and personal commitment can vary [37]. Hunter examines a similar phenomenon in South Africa among participants involved in self-defined girlfriend-boyfriend relationships where gifts are exchanged for sex and are “part of a broader set of obligations that might not involve a predetermined payment” [38]. Similarly, in sub-Saharan Africa, much sex outside commercial sex entails sex-for-money exchange which represents prescribed social norms in the form of gifts rather than identification as commercial sex [39]. Furthermore, sex work is often temporally fluid [40], as “women sometimes mix sex work with other economic activities and move in and out of it over time” (p. 207).

Looking back in time, some forms of prostitution in colonial Nairobi mimicked marriage, except that the women exchanged for money the domestic, emotional, and sexual services most wives at the time perform for free. According to White [41], many of these sex workers “were petty-bourgeois women who actively controlled profit-generating enterprises - the sale of sexuality, the sale of domestic skills, the rental of rooms, or all three-for which they provided the labor” (p. 175). White also notes that in colonial Nairobi, “customers occasionally became boyfriends or even husbands” and were apt to lend a hand to their sex worker partner when she had problems with a difficult client (p. 57). Some colonial sex workers also shared rooms and work routines, took care of each others’ children, paid each others' fines, and cooperated to protect and comfort each other, challenging the heteronormative notion of sex workers’ intimate relationships. These female friendships even had a harm reduction component as they made it possible for workers to have fewer customers. McClintok [42] notes that the Nairobi sex worker “exists at the flashpoint of the gendered division of money, sexual pleasure and labor, at the flashpoint of marriage and the market” (p. 99).

#### Rationale

These various studies suggest that the meshing of intimacy and sex work in well-resourced as well as less-resourced countries is much more than a site of victimization. Low income, high unemployment, single parenthood, the HIV crisis, and social deprivation continue to lead some women to engage in sex work in countries such as Kenya [43]. Chege et al. [44] argue that sex work remains a strategic employment choice for Kenyan women with dependents when there are significant constraints on formal labour. Heavy economic requirements due to caring for a large number of children often mean that they engage in additional economic activities, such as hairdressing or washing clothes [45]. Our study presents data on the benefits and constraints of intimate partnerships from a convenience sample of HIV+ sex workers residing in the urban slum of Kibera.

## Methods

### Study site

Kibera epitomizes the continuing enormous rural–urban migration in sub-Saharan Africa that has resulted in more than 50% of urban Kenyans now living in informal settlements. Indeed, it is habitually the first stop for people migrating to Nairobi [5,6,46]. Kibera lies in the South West of Nairobi City, just 7 km from the city centre. It is the largest informal settlement in East Africa, with population estimates ranging from 200,000 to 800,000 people living within one square mile. The area was uninhabited until the 1920s, when it was awarded to Sudanese Nubian soldiers who fought in the Great War [47]. The name Kibera originally meant “swamp” in the Nubian language, referring to the wet marshlands in the locale. The British Colonial government did not give property titles to residents and, consequently, most people in Kibera do not own their own land or houses [48]. This unstable access to land leaves residents exposed to even higher levels of poverty, resulting in fewer resources to help support themselves or their families. Kibera lacks paved roads, and most houses are made from mud and thatched with iron sheets. Clean water is scarce and expensive. Lacking any public sewage and refusal disposal, residents use communal pit latrines or improvised toilets.

Due to these unfavourable conditions and a lack of proper health services, poor health is a major social concern. Malnutrition rates are high and residents carry a heavy disease load [49]. Tuberculosis and HIV/AIDS are the leading causes of death for Kibera residents over the age of five [50]. The HIV/AIDS prevalence for adults aged 15–49 in Kibera is 12% compared to the national average of 6.3% [12]. HIV/AIDS prevalence for Kibera FSWs is even higher, at 27.2% [51].

Yet, Kibera has positive aspects which are often overlooked. This urban area is a source of cheap rent relative to the rest of Nairobi and attracts many small businesses which offer informal employment to people who are largely excluded from formal avenues of employment in Nairobi and neighbouring cities. Kibera is also home to several organizations working to improve living standards for local residents. One such organization, Maji na Ufanisi (Water and Effectiveness), focuses on water and sanitation issues to help rally the community to address wider socio-economic issues related to poverty, and encourage community mobilization.

### Data collection and analysis

*A Kenya Free of AIDS: Harnessing interdisciplinary science for HIV prevention* (KEFA) is a United States’ National Institutes of Health-funded Center Grant (R24) linking the University of Nairobi, Kenya, with the University of Washington, USA, and the University of Victoria, Canada. Along with infrastructure and training components, KEFA features four field-based pilot projects. Data for the analysis below came from one of these projects – Project 4, titled *Exploration of Kenyan Female Commercial Sex Workers and Their Male Partners- Life Course and Harm Reduction Approaches*. Project 4 is focused on FSWs and their families residing in Kibera and involves 3 phases of data collection: 1) adult female sex workers and a comparison group of other female workers in Kibera; 2) female sex workers and their clients; 3) female sex workers’ views of their intimate relationships. We have published results on phases 1 and 2 [12,51]. Here we report on phase 3 results.

Women in phase 3 were recruited through the female sex worker peer-leader system facilitated by the Centre for HIV Prevention and Research at the University of Nairobi. Participants were deemed eligible if they were currently working as sex workers in Kibera, were HIV+ and between the ages of 18 and 45 years. We restricted our sub-sample to sex workers who identified as HIV+ because of their health vulnerability and within this age range representing reproductively active women. In order to study intimate relationships across this life span, the sample was stratified by age, with ten women in each of the three age categories: 1) 18–24; 2) 25–34; 3) 34–45. Peer leaders from each of Kibera’s ten culturally distinct villages recruited women in order to get varied responses from different cultural backgrounds. Women were not required to have a current intimate partner to participate in our study but were informed of the purpose of our study before they agreed to take part. A sample of thirty participants was recruited. At this point the research team agreed no new information was emerging (i.e., we felt we reached saturation). All but three women were either currently in an intimate relationship or had been in an intimate relationship while making a living from sex work. If the women had recently been or were currently in more than one intimate partnership (only one participant said she was involved in more than one intimate relationship), we asked them to answer questions in regards to the intimate partner to whom they currently felt closest.

Interviews were conducted using a concurrent mixed-methods approach and included open and closed-ended questions in the same research instrument. Data collection was considered integrated at this stage because qualitative and quantitative data were collected simultaneously [52]. Choosing a mixed-method approach study increased the range and breadth of the research [53,54]. Canadian researchers, their English-speaking research assistants (RAs) and Kenyan RAs worked together for two weeks in a group setting to develop a culturally sensitive, relevant research instrument. Important terms were discussed at great length so that the Kenyan RAs, who also acted as translators and interviewers, would have a common understanding of key terms.

A few English terms proved difficult to understand by the local RAs. One such term was ‘common law’ relationship, which in Canada, for example, has become formalized and carries with it legal (as well as moral consequences) for intimate partners and their children. Our Kenyan research assistants felt the term did not make sense in Kenyan society and culture. They said that Kenyan people become intimate and sometimes they live together --‘wewe kuja, wewe kukaa’ (you come you stay)--but there are no legal (common law) implications to this relationship. Much discussion also ensued around the meaning of the terms ‘intimacy’ and ‘intimate’ partner. In the end, there was much more similarity than difference in our common understandings of these terms, in both cases approximating the emergent international definition discussed above in the research literature – i.e., intimate relationships remain constituted concurrently through affective and transactional relations; economic transactions, fundamental to the functioning of households and ongoing negotiations between couples, remain integral to intimate partnerships. Pretesting and revising the research instrument and performing mock interviews made the group more comfortable with cultural meanings of study terms and allowed us to identify further discrepancies or culturally inappropriate words. Kenyan RAs also translated the questionnaire into Kiswahili, so that both English and Kiswahili copies were available for the interview. Interviews were conducted over the course of three days in July, 2011.

Participants met the research team at the Salvation Army church of Kibera, a site jointly chosen by the Centre for HIV Prevention and Research staff and the peer leaders. The Salvation Army church was one of the first religious organizations in Kibera to open its doors to FSWs and several participants reported that they felt comfortable at the church because it had been welcoming to them over the years. Interviewers consisted of three teams of two women. Each team had one Kenyan RA and one English-speaking researcher (the first author) or one of her two English-speaking RAs. For the three days we were in the field, the Kenyan RAs circulated so that they were paired at some point or other with the three English-speakers, helping to reduce potential for interviewer bias. Before the interview began, the Kenyan RA introduced the Canadian team member and obtained permission from the participant for the English speaker to participate in the interview. One major concern during data collection was our identities as outsiders interviewing a vulnerable population with a history of colonialism. Kovach [52] urges researchers to remember that “critically reflective self-location is a strategy to keep us aware of the power dynamics flowing back and forth between researcher and participant” (p. 112). None of the woman declined our request to participate; in fact, many told us they felt that we had a ‘bond’ in talking about intimate partnerships because of our status as women. A common phrase when talking about their intimate partners or children was “well, you understand; we’re all women”.

Before the interview began, participants had the choice of conducting their interview in English or Kiswahili. The Kenyan RAs conducted the interviews, which were recorded, and English-speaking counterparts wrote down participants’ responses on the interview questions.

The interviews took from half an hour to an hour and a half to complete. The shortest interviews were the three women who had never had an intimate partner while they were sex workers. In this case, we gathered demographic information and only asked the final question that focused on their definition of an ideal intimate partnership. At the conclusion of the interview participants were encouraged to ask questions about the study or interviewers, and an honorarium of 500 Kenyan shillings was provided to demonstrate appreciation of their time and knowledge shared. During the interview, we provided participants with snacks and soda, both for themselves and to take home for their children.

Responses to closed-ended questions were entered and analyzed using SPSS 12.0 software. The qualitative data were analyzed using the following procedures: using a thematic analytical approach, one of the authors initially coded the answers to the questions relating to intimacy and intimate relationships, and the first author repeated this exercise and independently identified the central themes in the transcribed answers to our questions of interest. Based on an examination of the preliminary themes they arrived at independently, they drew a third list of common themes by relevant question. The first author then reviewed the list of themes and a final version was made. Transcriptions were subsequently coded thematically and compared for coding consistency/reliability. The qualitative results below present the dimensions of intimate partnerships based on this thematic analysis of participants’ responses.

The research was approved by the institutional ethics committees at the University of Nairobi/Kenyatta National Hospital, University of Washington, and University of Victoria.

## Results

Results show that the majority of participants had completed some formal schooling: 40.7% completed primary school and 44.4% completed secondary school, and post-secondary education completion was rare, at 3.7%. Just over half the participants were never married, while five women were divorced or separated and three were widows. Participants had an average of four children living in their household. One woman had 10 children living in her household, while other participants had between one and six children. Participants were involved in sex work for an average of 6.25 years, ranging from six months to 25 years. The majority of the sample had been involved in sex work for under 10 years. Participants averaged 1788.33 shillings (approx. $20 USD) per week from sex work, with only four women making over 2000 shillings (approx. $23 USD) per week and almost 45% earning less than 1000 (approx. $12 USD) shillings per week. Half of the participants held secondary employment which contributed an average of 634.62 shillings (approx. $7 USD) per week to their household income. Of these women, seven earned 400 shillings (approx. $5 USD) per week or less in their secondary occupation.

According to participants, all intimate partners completed some form of formal schooling. Over half had completed secondary education and just under one-quarter had completed college or university education. The vast majority of intimate partners were employed in the work force. Participants most frequently reported that their intimate partners were mechanics or manual labourers. Three women who were currently in relationships and one woman who was not currently in a relationship reported that their partners were female. In addition, six participants reported that their intimate partner was married to another person. Of these intimate partners, five were male and one was female. Overall, it appears that participants were more socially-disadvantaged than their intimate partners, but both groups faced formidable economic and social challenges.

Table 1 presents background data on FSWs currently in an intimate partnership compared to those who were not in a relationship but have been so in the past (excluding the three participants who never been in an intimate relationship). The data indicate that participants involved in an intimate relationship at the time of interview were older on average, lived a shorter time in Nairobi, had fewer biological or adoptive children in their household, lower personal incomes, significantly fewer clients in the past week, are much more likely to reveal their HIV+ status to their intimate partner, are almost twice as likely to have told their intimate partner that they are a sex worker, and are appreciably less likely to be emotionally, physically or sexually hurt by their intimate partner. Despite this variation, none of the differences between the two groups were statistically significant. There was little difference between the two groups in regard to marital status, educational achievement, years in sex work, and whether they revealed their HIV status to clients. In response to the question, “what is (or was) the gender of your intimate partner”, four participants answered female, while the rest answered male.

**Table 1:** Currently in an intimate partnership versus not currently in an intimate partnership

	**Currently in relationship**	**Currently *not *in relationship**
	n=15	n=12
**Age**	30.4	26.17
**Years in Nairobi**	14.6	16.75
**Income (Kenyan Shillings)**	1960	2690
**Children in household**	3.1	4.7
**Marital status**		
*Single never married*	9	7
*Divorced separated*	3	2
*Widowed*	3	3
**Education**		
*None*	2	1
*Primary*	6	5
*Secondary/A-level*	6	6
*College/ mid-level*	1	0
**Years in sex work**	6.34	6.11
**Number of clients in average week**	6.6	7.33
**Number who tell clients that they are HIV+**	6	5
**Number who have told partner they are HIV+**	12	6
**Number who have told partner they are a FSW**	7	3
**Number ever emotionally hurt by partner**	9	10
**Number ever physically hurt by partner**	5	7
**Number ever sexually hurt by partner**	3	6

We also asked our participants to tell us what types of support they receive in their current relationship or received in their most recent intimate partner relationship (see Table 2), and to explain their answers for the different types of support they mentioned. Again, while responses varied between groups, none were statistically significant. Four types of overlapping supports – economic, emotional, health and childcare – emerging from our thematic analysis are presented below.

**Table 2:** Comparison of current partner and Ex-partner types of support

**(Does/did) your partner support you…?**		
	**Currently in relationship (n. = 15)**	**Previously in relationship (n.= 12)**
**Financially**	10	7
**Emotionally**	9	6
**In terms of health and safety**	7	4
**With childcare**	6	5
**In other ways**	3	1
*Total support (average)	2.33	1.92

*Based on total number of questions to which participants answered “yes”.

### Monetary support

The majority of participants reported that their intimate partner provided them with some kind of monetary support. These participants discussed how their partners would bring them money when they received a paycheck, or upon direct requests for financial assistance. One woman, Helen [1], age 27 with 10 children in her household [2], described how she and her intimate partner kept their money together in order to share household expenses: “[I]f we join hands as the casual labourers [we] could help each other maybe pay rent by living together”. Intimate partners also contributed financially by buying household goods and food. Stellah, age 33 with 2 children, described how her partner went shopping about once a month for “household things, like soap”, in addition to sending her money. Partners also sometimes contributed financially to the well-being of the women's children. In one instance, Sharleen, age 31 with 3 children in her household, described how her partner would send money for her children: “[Sometimes he sends me money like 2000, 1000 [for the children], yeah”. In another instance, Eva, age 20 with 4 children in her household, described how her intimate partner assisted with her younger sister's school fees: “So, when the children are away from school he tries his best to get the school fees balanced so that they get back to school”.

At the same time, a number of participants, particularly those not currently in an intimate relationship, explained that their intimate partner did not offer enough monetary support or that such support was rare. Emily, age 33 with 6 children living under her roof, commented that her partner only supplied her with flour “for making ugali [a porridge-like dish made from maize flour] but nothing else with money”. Sharleen, age 31 with 3 children in her household, also described the financial support she received as insufficient: “Sometimes he gives it, like that only comes like once a week. He gives me even 1000 (Kenyan shillings). That is not—it’s not helping—it’s not helping me”. Some intimate partners offered financial support only occasionally. Lily, age 20 and 5 children in her household, mentioned that when she calls her intimate partner and tells him she has a problem he will send some money “but he’d never do it, you know, out of his initiative”.

In addition, several participants, especially those currently without a partner, stated that being in an intimate relationship resulted in *reduced* household income because they were less able to meet with paying clients while they were with their intimate partner. Felicity, age 18 with 5 children in her household, lamented: “For example, if he insist on you spending time with him in the house you can’t go out and work as a sex worker and the children don’t have food. So that’s a very big disadvantage”. Violet, age 35 with 1 child in her household, had a similar response: “Yeah, there are disadvantages because the intimate partner always wants you close to him. Maybe like now he doesn’t support me and wants me to be there”. Georgina, age 20 with 5 children in her household, elaborated that if I “was to go out every day with different clients [I] would have my own money… [W]ith an intimate partner you don’t get as much as you can, like you do with clients. This person [intimate partner] is not your husband so he’s not really dedicated to giving you everything”.

### Emotional support

Many participants reported that their intimate partners provided them with another dimension of support -- emotional care; again, women currently in an intimate relationship were more likely to report that their partners sustained them in this way. Some women, such as Tabitha, age 19 with 2 children in her household, discussed how their intimate partner comforted them when they were experiencing stress, including giving advice and telling them to relax and “not think so much about stress”. Other women commented that they could rely on their intimate partner for advice. Helen, who was quoted above, noted that her partner “used to help her [during] hard times; she could consult”.

Participants currently in an intimate relationship were likewise more likely to share their HIV status with their partners. Some intimate partners knew about the woman's HIV status before the relationship began and a small number of participants met their intimate partners while they undergoing treatment at clinics. Other participants talked of informing their partners of their status only to have their partners reveal their HIV positive status. In a couple of instances, when participants shared their status, this motivated their intimate partners to undergo HIV testing as well.

For Georgina, mentioned above, having a partner who shared “her” or “his” status resulted in a more emotionally supportive environment. Georgina and her HIV positive partner support each other and even attend HIV training groups together. Several participants also stated that their partners gave them hope and encouraged them by reminding them they were “not alone, that there are many other sick people and there's more to life than that” (Maria, age 25, 5 children). Some women, including Janet, age 37 with 6 children in her household, found that their status provided them with common ground with their intimate partners in which they could mutually provide support: “We wanted to live together as a couple who was sharing the same status, as well as same challenges”.

While several women talked of relationships that provided emotional support, mentions of romantic love were rare. Only two participants discussed romantic love, and both of these were currently in intimate relationships. Eva, age 19 with 1 child in her household, discussed how her income from sex work was reduced because of her intimate relationship but that she did not consider it a disadvantage: “When you love somebody, you don’t even love them because they have money or they don’t; you just find yourself loving them. So whether he has money or he doesn’t, it’s good if he’s around than if he’s not”. In another instance, Angy, age 37 with 2 children, explained how her partner wanted to continue the relationship after finding out she was HIV positive because he loved her.

While the majority of partners were emotionally supportive, especially for women currently in an intimate partnership, some had partners who were emotionally controlling or abusive. Joy, age 36 with 1 child in her household, explained that: “Having an intimate partner, he won’t let you be free; he really close you up and he won’t let you go out there”. Maria, mentioned above, described how her partner used her involvement in sex work as a way to guilt her into doing what he wanted: “At times I am forced to do what I don’t want; [he] wants to sleep with me and I just don’t feel like”. Maria stated that she gives in to her partner’s request “even when in anger”.

Participants who were not currently in an intimate relationship were more likely to report that their past intimate relationship was a source of stress or their past partner had been emotionally abusive. When asked if her partner provided emotional support, Sarah age 24 with 4 children in her household, responded, “He was a quiet guy. Only when he got drunk and he just abuse and talk rude”. One participant stated that her partner often called her a “person coffin” in response to her HIV+ status. Several women described how their intimate relationships caused them stress. Susan, age 25, with 2 children in her household, put it like this:

Okay, to me, I know this because, when I was just alone I was so happy, at least because nobody’s quarreling with me, as you know being HIV and AIDS, being positive. You’re not supposed to be having stress most of the times. So to me, it was not so good to me having an intimate because at the end of the day he just come and quarrel with me; he’s drunk, maybe he wants to fight with you. So it’s just like no, let me just be alone.

Other sources of stress identified by women who were no longer in an intimate partnership included: physical and sexual violence, emotional abuse, poor treatment of their children by their partner and violence connected to the dissolution of the relationship.

### Health support

As noted above, participants who were currently in intimate relationships were more likely to tell their partners about their HIV status than those women who were not currently in an intimate relationship. Not surprisingly, they were also more likely to receive support for taking their anti-retroviral (ARV) medications. Several participants currently in intimate relationships talked about how their partners bought their medications when they needed them: “If I go to the hospital, and maybe I’m prescribed a drug, can buy for me. Yeah, he helps me” (Violet, age 35 with 1 child in her household). In one instance, Faith, age 25 with 6 children in her household, explained that both she and her intimate partner were HIV positive and shared the responsibility of travelling to the clinic for ARV medications. Some intimate partners also reminded women to take their medications on time. As Maria explained, her partner uses his cell phone to remind her even when they were not together.

Participants currently in an intimate relationship also talked about how their partners encouraged a healthy lifestyle and positive living through drinking less, eating better food and exercising. One participant, Becky, age 21 with 1 child, stated that her partner went out of her way to bring her food. Violet's partner inspired a healthier lifestyle by encouraging her to drink less: “He’s helped me stop taking alcohol. Okay, I’ve not stopped but I’ve reduced”. Sue, age 36 with 1 child, noted that she and her partner reminded each other to drink the medication, to exercise and to eat well”.

### Child care support

A final dimension of support provided by intimate partners was help with the women’s biological and adopted children. Janet's intimate partner helped administer her young child's HIV medications when she needed to go out alone and Felicity's partner would come to her house to help educate her children. Eva's partner acted as a father figure by encouraging the children to avoid getting into trouble. “So in terms of children he also advises them, like they should come from school and go home straight always to avoid getting into mix up with strangers”. Participants with female intimate partners were more likely to receive childcare support. Mary, age 21 with 5 children in her household, said her partner assisted by giving her childcare advice. Mary also noted that when she had a problem, she could confide in her intimate partner. For Grace, age 20 with 3 children, her female partner helped guide her when she had issues. Mary’s partner was also a sex worker and sometimes cared for Mary’s children when she was providing sex services to paying clients.

However, the majority of participants – whether talking about their current relationship or a past one -- reported that they did *not* receive childcare support from their intimate partners. Rebecca, age 32 with 5 children in her household, mentioned her intimate partner was not willing to provide childcare, with the rationale that he is not the biological father. In addition, some participants felt that they would be better off ending their intimate relationships to focus on caring for the children in their household. Though Eve's intimate partner asked her to get married and offered to help her start a non-sex work business, she remained reluctant because of her continuing feeling of responsibility to raise her younger sisters’ children. Anne, age 32 with 4 children in her household, found herself in an even worse situation and eventually ended contact with her intimate relationship because he was mean to her children: “Once he put washing powder, like laundry soap, in the drinking water, so the children drank the water and got sick. Then most of the time he would come home [drunk] and insist on [us] having sex, even when the children are there”.

### The ideal and the reality

Many participants described their ideal intimate partner as someone who would help provide financial support. These women wanted a partner who could help them meet their financial obligations, such as rent and school fees, and several women expressed hope that financial support would enable them to leave sex work:

An intimate relationship should be where the man provides for you, provides for your needs. He should pay your rent. He pays your rent, takes your kids to school. He can be concerned about where you get your breakfast, your lunch. (Angy, age 37, with 1 child in household).

Some participants felt that emotional support, such as offering comfort and providing care and understanding for participants' HIV status, played a more paramount role: “When you have someone who is human [and] able to help you when you’re down. Help in maybe with making your medicine when you’re not able. And again, someone who would be there for you because living alone when you have this disease makes you feel so lost—you’re down, you’re all alone” (Helen, age 27, with 10 children in household).

The majority of participants reported that both financial and emotional support were important for an ideal partnership. Stellah, age 33 with 2 children in her household, stated that she wanted a partner that could be caring and assist her both financially and emotionally and show understanding for her HIV status: “He is caring, he can provide for my needs like—can tell him everything. Mmhmm. He can understand and agree with my status. An ideal”. Susan, age 32 with 2 children in her household, described how her past relationships had helped her form an idea of the ideal partnership that would be emotionally supportive and eventually lead to her exiting sex work: “Okay, to me if I get an intimate partner now, since I’ve learned a lot, I would like to have an intimate partner who’s supportive, caring, and maybe at the end of it we get married so I would stop this sex work and I have my family and live a happy life”. Some women also mentioned an ideal partner would not stigmatize them for doing sex work, respect them, and not put them down in front of their children because they accepted money from sex clients.

Many women, especially those who were not currently in relationships, also expressed a desire to have an intimate partner who would help support their children by taking them to school or providing for them financially Charity, age 24 with 4 children in her household, also wanted a partner who would treat her children well: “Someone who can be good to my kids, someone who can provide us food, clothing, shelter”. Participants likewise felt that ideal partners should care about their health status. Sue, age 31 with 1 child, talked of an ideal partnership where her partner assisted with her HIV care: “And he should help you in terms of health. He should remind you how to take your medication and when you are supposed to go for your clinic and he should take you”.

Finally, a number of participants identified commitment and mutual support as being important facets of an ideal partnership. Gina's vision of an ideal relationship included not only financial support but commitment: “Someone who is not married or does not have a wife out there. Someone who doesn’t have any other commitment to any woman out there”. Joy, age 36 with 1 child, felt that mutual support was important in an ideal relationship: “He helps you when you have trouble. When you have trouble he won’t leave you. And when he also has problems you won’t leave him. ” For nearly half of the participants who are currently involved in an intimate partnership, having a partner gave them an opportunity to think about a different future. Participants talked of living with, marrying, or starting a business with their intimate partner. One participant, Becky, age 21 with 1 child in her household, had hopes to start a clothing business with her partner in order to leave sex work and spend more time with her child.

Five participants felt that their relationships did not have a future because their partners were already married. Being in a relationship with a married man resulted in women reporting an uncertain future with their partners. Some women, such as Maria and Rebecca, stated that they could not make future plans with their partners because their partners’ marriages meant that the relationship could not continue long term or that “it can end at any time”. Sharleen, age 31 with 3 children, explained: “When he comes, or when we meet he just gives me some, like 2000, then he tells me, you know you’re not my real wife and I’m just assisting you”. Similarly, Susan, age 35 with 2 children, and Serah, age 24 with 4 children, both discussed how they felt their partners were using them. In another instance Karen, age 20 with 2 children, explained that her relationship had no future because of her partner's drinking:

Interviewer: And did you have any plans…

Karen: I would go to his place or he would come to my place. We had plans but no you see this man he would just drink and every day, every day, so…just…

Interviewer: He drank every day?

Karen: Yeah. So there’s no future there. So it is better I do my work and concentrate on my things and leave him alone.

## Discussion

While some scholarship on intimacy in ‘late modern’ well-resourced societies argues that it is more and more divorced from instrumentality [21], studies in both well-resourced and resource-constrained countries around the globe provide convincing evidence that intimate partnerships are seldom free from transactional concerns; rather, economic and affective elements are bonded within intimate relationships [25]. Other studies have recently made the same point in regard to intimacy and sex work, which has dimensions that range from the transactional to the intimate [26,27]. This finding has important implications for understanding possible benefits of intimacy partnerships in countries such as Kenya where low income, high rates of unemployment and single parenthood, the HIV crisis, and social deprivation continues to lead some women to engage in sex work [39,43] and may even be a calculated employment choice for Kenyan women with dependents when there are significant constraints on formal labour.

Although intimacy is simultaneously constituted through affective and transactional elements, it is in how these elements are interwoven in particular times and places [23], and, what implications intimate relations have for the economic, social, and physical well-being of FSWs and their children that is of utmost interest [55]. The most significant theme that emerges from our study is that participants experienced intimate relationships in a range of ways, and both positively and negatively. In fact, our findings highlight the complex manner in which intimate and transactional relations are bound with one another, and, more specifically, how these intersect with the structural realities and vulnerabilities that mark the lives of the women we interviewed. An example of these unique intersections can be seen in how intimate partnerships tend to mean both a reduction in clients and a lower income, so that these partnerships can provide, simultaneously, some health protection, while also increasing risk of economic vulnerability and dependence. Indeed, the fact that there may be an economic *cost* associated with being in some intimate partnerships is highly revealing of how the structural inequities faced by Kibera FSWs have implications in terms of their ability to negotiate supportive partnerships.

In short, our findings suggest that the intimate relationships of the participants in our study cannot truly be understood in the terms of affective versus transactional elements. Instead, it is more fruitful to examine how intimate partnerships of FSWs exist on a continuum of risk and support, of benefits and constraints. Seen from this light, the transactional, instrumental, as well as the affective elements of intimate relations are equally implicated at both ends of the continuum, that is, whether the relationship provides monetary, emotional, health and childcare supports [2,28,29,56,57], or adds economic and health risks for themselves and their children [33].

It is also important to note that this entanglement of emotional and economic costs and benefits of intimate relations is not unique to intimate partnerships of FSWs in Kibera, Kenya. What our findings do emphasize instead is the overall significance of economic and social vulnerability to the intimate experiences of people, over and above the risks associated with working in the sex industry. For instance, both poverty and economic vulnerability, at the community and individual levels, are consistently linked with higher risk of partner violence in well-resourced nations like the United States and Canada [58]. Similarly, the fact that minorities and recent migrants within these countries also face higher risks of marital dissolution reminds us of how vulnerability is a key stressor on intimate partnerships [59]. In other words, it is possible that the defining feature of the intimate lives of FSWs is not that they are sex workers, but rather, that they are economically, socially, and physically vulnerable because of the multiple intersecting disadvantages they face [60].

In addition to this, however, the central implication of this research is that intimate relationships are an important determinant of the well-being of FSWs. As such, they are worthy of being the focus of not only further research, but also of community-based interventions aimed at supporting healthy intimate partnerships for HIV+ FSWs. Several of the participants, for instance, were clearly receiving help and support to improve their health; intimate partnerships for FSWs are thus a potential resource for improving treatment adherence. In addition to interventions at the interpersonal level, our results suggest the need for different policies and programs that support FSWs’ empowerment at the community level. Our findings do not support the abolitionist perspective that suppresses sex workers’ agency and daily struggle to make choices for themselves and their children, albeit within the structural constraints within which they live their lives. Depicting these women as vectors of disease and generating punitive policies intended to abolish the commercial sex sector will do little in terms of empowering sex workers to avail themselves of the resources available to them, including supportive intimate partnerships, and reject those that will worsen their health and that of their families [14,15,61]. It is more fruitful to better comprehend the intended and unintended effects of the structural environment on the health of sex workers [62].

## Conclusion

There is scant research on FSWs' intimate partnerships world-wide. The few studies done have focused on the constraints of such relationships, including the low level of condom use and high levels of physical violence between couples, as well as the risk of HIV transmission. Building on research from a previous field season of the Kenya Free of AIDS project, our study explores the benefits and constraints of Kibera FSWs’ intimate relationships in one informal urban settlement in Nairobi, Kenya.

This paper is not without limitations. It is based upon a comparatively small convenience sample, and therefore results cannot be extrapolated to other Kibera sex workers or local women not engaged in sex work, or other populations and/or settings. In addition, responses to our interview questions may be subject to social desirability bias. Nonetheless, these findings support the small number of previous studies highlighting the potential supportive role intimate partnerships can play to increase the health and safety of sex workers facing HIV, heavy childcare loads and other formidable odds.

## Endnotes

^a^Pseudonyms are used to protect the identity of the participants.

^b^This number includes biological and adopted children.

## Competing interests

The authors declare no competing interests concerning this paper.

## Authors’ contributions

All of the authors planned the study, and the first author and sixth author helped to conduct the interviews and generally oversaw the data collection. All authors contributed to the data analysis, drafting of the initial manuscript, and read and approved the final version.
